# Association between metabolic dysfunction‐associated steatotic liver disease and myosteatosis measured by computed tomography

**DOI:** 10.1002/jcsm.13543

**Published:** 2024-07-16

**Authors:** Myung Jin Kim, Yun Kyung Cho, Eun Hee Kim, Min Jung Lee, Woo Je Lee, Hong‐Kyu Kim, Chang Hee Jung

**Affiliations:** ^1^ Department of Internal Medicine Asan Medical Center, University of Ulsan College of Medicine Seoul Republic of Korea; ^2^ Asan Diabetes Center Asan Medical Center, University of Ulsan College of Medicine Seoul Republic of Korea; ^3^ Health Screening and Promotion Center Asan Medical Center, University of Ulsan College of Medicine Seoul Republic of Korea

**Keywords:** metabolic syndrome, sarcopenia, skeletal muscle, steatotic liver disease

## Abstract

**Background:**

In 2023, the concept of metabolic dysfunction‐associated steatotic liver disease (MASLD) was introduced as an alternative to non‐alcoholic fatty liver disease (NAFLD). We aimed to assess the quantity and quality of skeletal muscle using each of these diagnostic classifications.

**Methods:**

This cross‐sectional study included 18 154 participants (11 551 [63.6%] men and 6603 [36.4%] women, mean age 53.0 ± 8.8). The participants were classified into four categories: neither steatotic liver disease (SLD), NAFLD only, MASLD only or both SLDs. An appendicular skeletal muscle mass adjusted for body mass index of <0.789 for men and <0.512 for women was defined as sarcopenia. The total abdominal muscle area (TAMA) at the L3 vertebral level was segmented into normal‐attenuation muscle area (NAMA), low‐attenuation muscle area and intermuscular/intramuscular adipose tissue. Myosteatosis was defined by a T‐score < −1.0 of the NAMA/TAMA index, which was calculated by dividing the NAMA by the TAMA and multiplying by 100.

**Results:**

Using subjects with neither SLD as a reference, the multivariable‐adjusted odds ratios (ORs) for sarcopenia were significantly increased in those with MASLD, with adjusted ORs (95% confidence interval [CI]) of 2.62 (1.94–3.54) in the MASLD‐only group and 2.33 (1.92–2.82) in the both SLDs group, while the association was insignificant in those with NAFLD only (adjusted OR [95% CI]: 2.16 [0.67–6.94]). The OR for myosteatosis was also elevated in the MASLD groups, with an OR (95% CI) of 1.75 (1.52–2.02) in subjects with MASLD only and 1.70 (1.57–1.84) in those with both SLDs, while it was slightly decreased in subjects with NAFLD only (0.52 [0.29–0.95]).

**Conclusions:**

Employing the MASLD concept rather than that of the NAFLD proved to be more effective in distinguishing individuals with reduced muscle mass and compromised muscle quality.

## Introduction

Metabolic dysfunction‐associated steatotic liver disease (MASLD) has recently emerged as a novel conceptual framework proposed to replace the traditional term non‐alcoholic fatty liver disease (NAFLD).^1^ This paradigm shift was introduced by esteemed international experts in 2023 to reflect the complex pathogenesis and underlying metabolic abnormalities associated with steatotic liver disease (SLD).^1^ The term NAFLD was to describe cases of SLD in non‐heavy drinkers with inflammation similar to alcoholic steatohepatitis. It is an embracing term that covers the full spectrum of simple fatty liver, non‐alcoholic steatohepatitis (steatosis with inflammation) and fibrosis/cirrhosis.^2^

NAFLD is diagnosed by exclusion, meaning that the coexistence of other liver diseases, such as viral hepatitis, autoimmune diseases or alcoholic hepatitis, is not considered when evaluating the progression of SLD.^3^ Despite the need for prompt intervention for individuals with both metabolic risk factors and concomitant alcohol use, no specific category has previously been available for their diagnosis. The new concept of MASLD is defined by a set of positive diagnostic criteria for SLD associated with metabolic dysfunction rather than by criteria based on exclusion.^1^ This change reflects the need to focus on metabolic factors in SLD, as the adverse outcomes of NAFLD are mostly associated with metabolic syndrome.^4^

The relationship between chronic liver disease and sarcopenia has been an increasingly popular topic of study. Sarcopenia appears in the early stages of liver disease and worsens as SLD progresses,^5^ with a prevalence rate of up to 60% in people with cirrhosis.^6^, ^7^ Myosteatosis, characterized by the infiltration of muscle with fat,^8^ has also been shown to worsen SLD and increase mortality in patients with cirrhosis.^9^, ^10^ While the impact of MASLD on various cardiovascular outcomes has been previously discussed,^11^, ^12^, ^13^ its implications for peripheral tissues, particularly skeletal muscle, remain unexplored.

Our study aimed to investigate the prevalence of SLD and evaluate the quantity and quality of skeletal muscle by adopting both the MASLD and NAFLD diagnostic classifications.

## Material and methods

### Study population

In this cross‐sectional study, 18 154 individuals who underwent abdominal computed tomography (CT) and abdominal sonography as a part of health screening exams at the Health Screening and Promotion Center of the Asan Medical Center in Seoul, Republic of Korea, between January 2012 and December 2013 were included. The potential harm of CT, including radiation exposure, was fully explained to the participants, and written consent was obtained from each subject before the CT scan.

Participants were excluded if they had any of the following: history of malignancy; history of cardiovascular diseases (myocardial infarction, congestive heart failure and cerebrovascular accidents); chronic kidney disease (estimated glomerular filtration rate [eGFR] < 60 mL/min/1.73 m^2^); overt or subclinical thyroid dysfunction (free T4 of >1.9 or <0.8 ng/dL and thyroid‐stimulating hormone of <0.4 or >5.0 mU/L); current glucocorticoid replacement; current sex hormone replacement; alcohol intake meeting the criteria for alcohol‐associated/related liver disease (>350 g/week [50 g/day] for females and >420 g/week [60 g/day] for males); or missing data.

### Laboratory and anthropometric assessments

We used standardized questionnaires to collect sociodemographic and lifestyle information. Alcohol consumption was calculated as grams per day, and smoking status was classified as past‐, current‐ and never‐smoker. Regular exercise was defined as performing 30 min of moderate‐intensity aerobic exercise for ≥5 days/week, 20 min of vigorous‐intensity exercise for ≥3 days/week or resistance exercise for ≥3 days/week. The waist circumference, height and weight were measured by standardized protocols. Body mass index (BMI) was derived by dividing weight in kilograms by height in metres squared.

Laboratory analysis was performed by an authorized central lab at Asan Medical Center. Further explanations for the anthropometric and laboratory measurements are described in the Supplementary Methods section of the supporting information.

### Diagnosis of steatotic liver disease

SLD was diagnosed using abdominal ultrasonography (Ultrasound Systems IU22; Philips, Amsterdam, Netherlands). Abdominal ultrasonography was conducted by expert radiologists who were unaware of the study participants' laboratory and clinical features. SLD was identified based on characteristic ultrasonographic findings including hepatic parenchymal brightness, contrast between the hepatic and renal parenchyma, vascular blurring, focal sparing and narrowing of the hepatic vein lumen.^14^

#### Diagnosis of non‐alcoholic fatty liver disease

NAFLD was diagnosed when abdominal sonography showed findings consistent with fatty liver, and both of the following were excluded: (a) secondary causes of liver disease and (b) excessive alcohol consumption (≥30 g/day for males and 20 g/day for females).^15^

#### Diagnosis of metabolic dysfunction‐associated steatotic liver disease

MASLD was diagnosed based on radiologically confirmed hepatic steatosis and the presence of any one of the criteria listed below: (a) overweight or obesity (BMI ≥ 23 kg/m^2^ in Asians) or waist circumference of ≥90 cm in men and ≥80 cm in women (following Asian criteria); (b) fasting plasma glucose (FPG) of 5.6–6.9 mmol/L (100–125 mg/dL), glycosylated haemoglobin (HbA1c) of 5.7–6.4% (39–47 mmol/mol) or the presence of diabetes mellitus; (c) blood pressure ≥ 130/85 mmHg or on an anti‐hypertensive drug; (d) plasma triglycerides (TGs) ≥ 1.70 mmol/L (150 mg/dL) or on lipid‐lowering therapy; and (e) high‐density lipoprotein cholesterol (HDL‐C) of <1.0 mmol/L (40 mg/dL) for men and <1.3 mmol/L (50 mg/dL) for women.

In our study, patients who met the criteria for MASLD or MetALD (MASLD and increased alcohol intake) were categorized as the MASLD group, as we aimed to emphasize the importance of metabolic risk factors in the clinical outcomes of SLD, irrespective of alcohol consumption.

**Figure 1:**
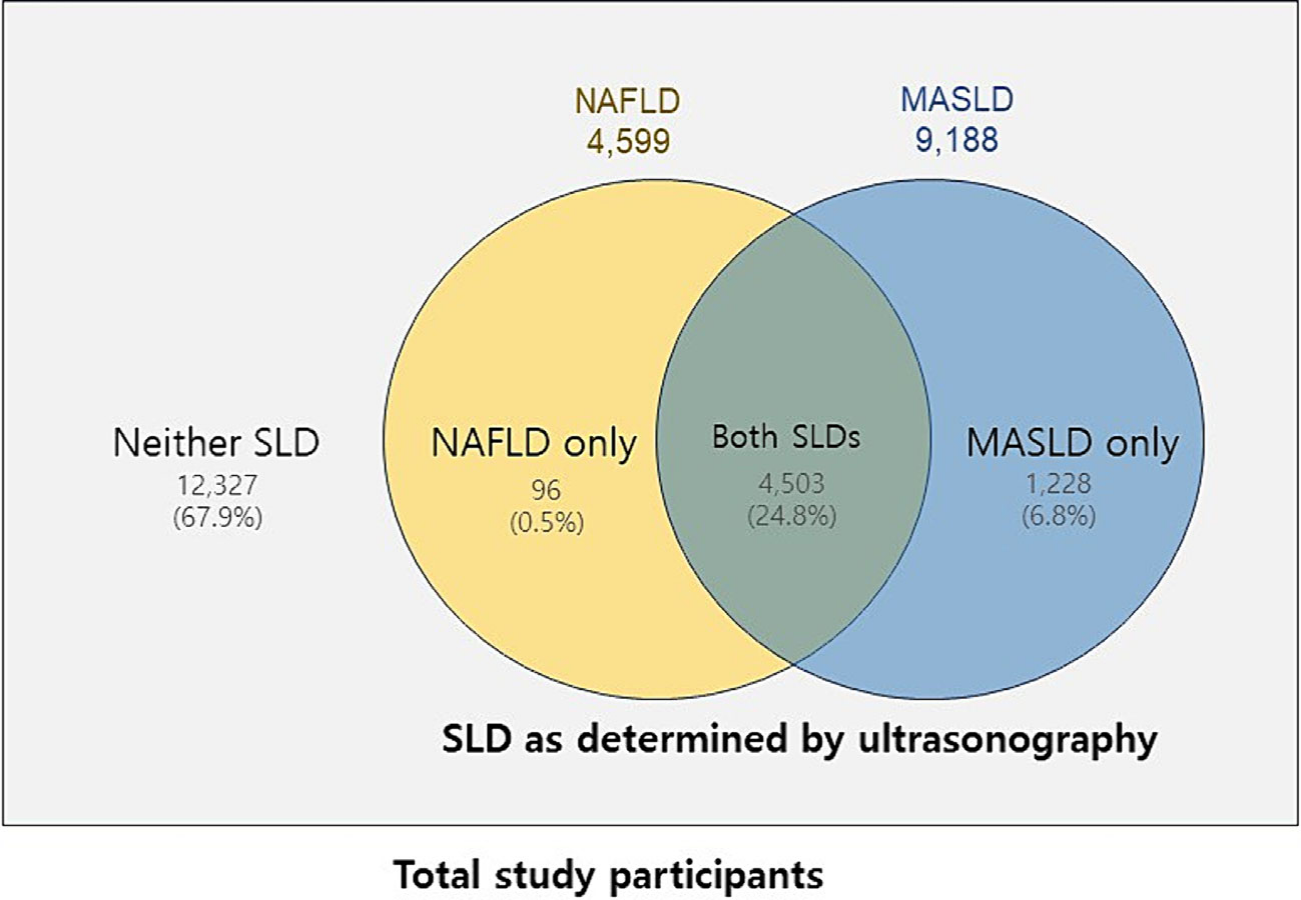
Grouping of study participants according to the presence of steatotic liver disease (SLD) and the diagnostic criteria for non‐alcoholic fatty liver disease (NAFLD) and metabolic dysfunction‐associated steatotic liver disease (MASLD).

In this study, the participants were classified into four categories: neither SLD, NAFLD only, MASLD only or both SLDs (*Figure* *1*).

### Evaluation of sarcopenia

Appendicular skeletal muscle mass was determined by the sum of the lean muscle mass of both limbs,^16^ measured with bioelectrical impedance analysis (InBody 720), as described in the Supplementary Methods section of the supporting information. We used the cut‐off values recommended by the Foundation for the National Institutes of Health to define sarcopenia^17^: appendicular skeletal muscle adjusted for BMI of <0.789 for men and <0.512 for women.

### Evaluation of myosteatosis

CT was performed according to a prespecified protocol, as explained in the Supplementary Methods section of the supporting information. The cross‐sectional area of the skeletal muscle at the L3 vertebral level was measured. Body composition was automatically interpreted from CT images using an artificial intelligence program with the segmentation technique of a fully convolutional network.^18^ The automatic system was used to segment the axial images into the subcutaneous fat area, visceral fat area and total abdominal muscle area (TAMA). The TAMA included all the muscles seen in the images, including the psoas, paraspinal, transversus abdominis, rectus abdominis, quadratus lumborum and internal and external obliques. *Figure* *S1* is a graphical description showing how the body composition in the axial CT image was analysed.

TAMA was further segmented based on mean attenuation densities (Hounsfield unit [HU]) on CT scan: (1) +30 to +150 HU into normal‐attenuation muscle area (NAMA), a muscle area with little intramuscular lipid; (2) −29 to +29 HU into low‐attenuation muscle area (LAMA), a muscle area with the fatty component; and (3) −190 to −30 HU into intermuscular/intramuscular adipose tissue (IMAT). The NAMA/TAMA index, which represents the degree of good‐quality muscle, was calculated by dividing the NAMA by the TAMA and multiplying by 100.^16^ In our study, myosteatosis was defined by a T‐score < −1.0 of the NAMA/TAMA index (<73 in men and <72 in women).^19^

### Statistical analysis

Continuous variables following normal distributions are presented as the mean and standard deviation (SD), while variables with skewed distributions are reported as medians and interquartile ranges. To assess the baseline characteristics among the SLD groups, we employed analysis of variance (ANOVA) with Scheffe's method for post hoc analysis and the Kruskal–Wallis test with the Dunn procedure for those with skewed distributions. Categorical variables were evaluated using a chi‐square test.

The odds ratios (ORs) and 95% confidence intervals (CIs) of each group for having sarcopenia and myosteatosis were analysed separately for men and women. Five models with different adjustment variables were used to demonstrate the risk analysis: the unadjusted model; Model 1, which adjusted for age; Model 2, which adjusted for smoking and exercise in conjunction with the factors included in Model 1; Model 3, which adjusted for eGFR and high‐sensitivity C‐reactive protein (hsCRP) in addition to the variables included in Model 2; and Model 4, which adjusted for the visceral fat area divided by subcutaneous fat area in addition to Model 3. Finally, we conducted a subgroup analysis according to the presence of general overweight or obesity (BMI ≥ 23 kg/m^2^), abdominal obesity (waist circumference of ≥90 cm in men and ≥80 cm in women) and the presence of diabetes mellitus in the MASLD group.

SPSS software Version 21.0 for Windows (IBM, Inc., Armonk, NY, USA) was used for all statistical analyses. Statistical significance was determined as a *P*‐value < 0.05.

## Results

### Baseline characteristics of the study population

**Figure 2:**
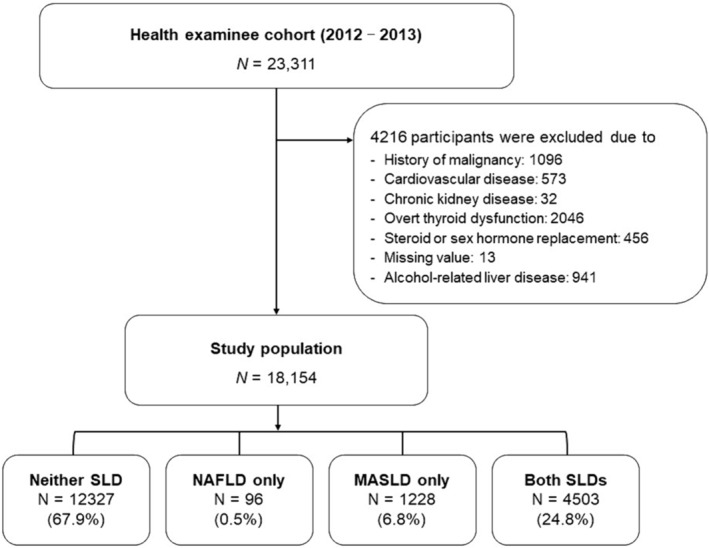
Study population. MASLD, metabolic dysfunction‐associated steatotic liver disease; NAFLD, non‐alcoholic fatty liver disease; SLD, steatotic liver disease.

A total of 18 154 participants (11 551 [63.6%] men and 6603 [36.4%] women) were included in the final analysis. The distribution of individuals with respect to the SLD subtypes was as follows: 67.9% had neither SLD, 0.5% had NAFLD only, 6.8% had MASLD only and 24.8% had both SLDs (*Figure* *2*). The mean age of the subjects was 53.0 ± 8.8 years, and the mean BMI was 23.9 ± 3.0 kg/m^2^.

The baseline characteristics of the study subjects according to the SLD groups are presented in *Table* *1*. Individuals with MASLD only and both SLDs showed generally unfavourable metabolic profiles such as lower HDL‐C and higher TG, FPG, HbA1c and Homeostatic Model Assessment for Insulin Resistance (HOMA‐IR) levels compared with those with neither SLD or NAFLD only. Subjects with MASLD only and both SLDs also had more appendicular skeletal muscle, of which the trend was reversed when adjusted for BMI. Regarding the CT measurements, the TAMA, NAMA, LAMA and IMAT were higher in subjects with MASLD only and both SLDs, but the NAMA/TAMA index, which refers to the amount of good‐quality muscle, was lower in those with MASLD only and both SLDs. *Table* *S1* shows a comparison of the characteristics of the participants based on the presence of NAFLD or MASLD.

**Table 1:** Baseline characteristics and computed tomography measurements of the participants according to the steatotic liver disease groups

Variables	Neither SLD	NAFLD only	MASLD only	Both SLDs	*P*
*N* (%)	12 327 (67.9%)	96 (0.5%)	1228 (6.8%)	4503 (24.8%)	
Age (years)	52.7 ± 8.9_a_	50.6 ± 7.1_ab_	51.7 ± 7.7_b_	54.2 ± 8.8	<0.001
Sex (male, *N* [%])	7224 (58.6%)	60 (62.5%)	1154 (94.0%)	3113 (69.1%)	<0.001
Body mass index (kg/m^2^)	23.0 ± 2.6	21.6 ± 1.1	26.1 ± 2.7	25.7 ± 2.8	<0.001
Waist circumference (cm)	81.5 ± 8.2_a_	79.5 ± 5.1_a_	91.6 ± 7.1	89.6 ± 7.6	<0.001
Systolic BP (mmHg)	120.1 ± 14.0	114.6 ± 8.1	128.3 ± 13.1	126.0 ± 13.5	<0.001
Diastolic BP (mmHg)	76.1 ± 10.7	72.6 ± 6.9	83.3 ± 10.1	80.0 ± 10.3	<0.001
Current smoker (%)	21.2	20.0	35.7	24.4	<0.001
Excess drinking (%)	23.1	0.0	85.2	0.0	<0.001
Physically active (%)	46.0	33.3	36.6	40.8	<0.001
Obesity (BMI > 25 kg/m^2^) (%)	21.4	0.0	65.1	55.7	<0.001
Hypertension (%)	6.7	0.0	20.0	19.6	<0.001
Dyslipidaemia therapy (%)	9.6	0.0	14.5	17.1	<0.001
Family history of diabetes (%)	20.7	20.8	28.7	25.2	<0.001
FPG (mg/dL)	97.4 ± 14.9	90.6 ± 6.0	107.8 ± 22.0	106.1 ± 22.7	<0.001
HbA1c (%)	5.51 ± 0.54	5.32 ± 0.21	5.86 ± 0.82_a_	5.91 ± 0.85_a_	<0.001
Total cholesterol (mg/dL)	193.5 ± 33.2_a_	198.4 ± 28.6_ab_	199.0 ± 36.7_b_	198.3 ± 37.0_b_	<0.001
TG (mg/dL)	90 (67–124)	92 (75–113)	142 (103–199)	130 (96–176)	<0.001
LDL‐C (mg/dL)	120.6 ± 29.7	129.5 ± 24.2_a_	126.9 ± 32.4_a_	128.7 ± 32.7_a_	<0.001
HDL‐C (mg/dL)	58.4 ± 14.8_a_	55.2 ± 9.3_a_	49.6 ± 11.7_b_	48.4 ± 11.5_b_	<0.001
Uric acid (mg/dL)	5.15 ± 1.35_a_	5.39 ± 1.17_a_	6.19 ± 1.35	5.77 ± 1.38	<0.001
AST (U/L)	27.0 ± 14.5_a_	25.9 ± 7.2_ab_	33.8 ± 37.8	29.6 ± 13.7_b_	<0.001
ALT (U/L)	22.6 ± 17.4_a_	25.9 ± 13.6_a_	36.4 ± 34.5	33.0 ± 22.1	<0.001
GGT (U/L)	18 (13–30)	19 (13–30)	40 (27–64)	26 (18–39)	<0.001
hsCRP (mg/L)	0.04 (0.02–0.08)	0.04 (0.03–0.10)	0.07 (0.04–0.14)	0.07 (0.04–0.14)	<0.001
HOMA‐IR	0.90 (0.54–1.41)	0.87 (0.58–1.43)	1.72 (1.19–2.44)	1.69 (1.13–2.42)	<0.001
Body fat mass, kg	14.8 ± 4.7_a_	13.7 ± 2.5_a_	19.2 ± 5.5_b_	19.4 ± 5.7_b_	<0.001
Skeletal muscle mass, kg	26.9 ± 5.7_a_	25.7 ± 4.8_a_	32.0 ± 4.6	29.0 ± 5.9	<0.001
Appendicular skeletal muscle, kg	20.3 ± 4.5_a_	19.6 ± 4.1_a_	24.1 ± 3.5	21.8 ± 4.6	<0.001
Appendicular skeletal muscle/BMI, m^2^	0.88 ± 0.17_a_	0.91 ± 0.18_ab_	0.93 ± 0.12_b_	0.85 ± 0.16	<0.001
Visceral fat area/subcutaneous fat area	0.78 ± 0.47_a_	0.83 ± 0.39_a_	1.30 ± 0.52	1.11 ± 0.52	<0.001
TAMA, cm^2^	142.6 ± 32.4_a_	134.9 ± 27_a_	175.1 ± 25.7	157.2 ± 32.9	<0.001
NAMA, cm^2^	109.6 ± 29.8_a_	106.2 ± 25.4_a_	132.5 ± 24.1	117.1 ± 31.3	<0.001
LAMA, cm^2^	27.8 ± 10.2	24.4 ± 7.7	36.2 ± 12.3	33.7 ± 11.6	<0.001
IMAT, cm^2^	5.12 ± 3.96_a_	4.28 ± 3.20_a_	6.42 ± 4.54_b_	6.49 ± 5.14_b_	<0.001
NAMA/TAMA index	76.5 ± 8.6_a_	78.3 ± 7.3_a_	75.6 ± 8.0	73.8 ± 9.4	<0.001

*Note*: The same subscripts imply a statistically insignificant difference between those values in post hoc analysis. Otherwise, post hoc analysis revealed significant differences between each group. Abbreviations: ALT, alanine aminotransferase; AST, aspartate aminotransferase; BMI, body mass index; BP, blood pressure; FPG, fasting plasma glucose; GGT, gamma‐glutamyltransferase; HbA1c, glycosylated haemoglobin; HDL‐C, high‐density lipoprotein cholesterol; HOMA‐IR, Homeostatic Model Assessment for Insulin Resistance; hsCRP, high‐sensitivity C‐reactive protein; IMAT, intermuscular adipose tissue; LAMA, low‐attenuation muscle area; LDL‐C, low‐density lipoprotein cholesterol; MASLD, metabolic dysfunction‐associated steatotic liver disease; NAFLD, non‐alcoholic fatty liver disease; NAMA, normal‐attenuation muscle area; SLD, steatotic liver disease; TAMA, total abdominal muscle area; TG, triglyceride.

### Risk of sarcopenia according to each steatotic liver disease group

*Table* *2* presents the prevalence of sarcopenia and the corresponding adjusted ORs with 95% CIs. In both men and women, the prevalence of sarcopenia was slightly higher in individuals with MASLD (5.2% and 6.0% in the MASLD‐only and both SLDs groups in men and 5.4% and 3.8% in women, respectively) than in those with NAFLD only (5.0% in men and 0.0% in women). In individuals with MASLD, the ORs for sarcopenia were significantly increased, with the multivariable‐adjusted ORs (95% CIs) of 2.38 (1.74–3.25) in those with MASLD only and 2.37 (1.91–2.95) in those with both SLDs among men and 4.72 (1.55–14.36) in those with MASLD only and 2.88 (1.85–4.49) in those with both SLDs among women (*Table* *2*, Model 4).

**Table 2:** Prevalence and odds ratios for having sarcopenia according to the presence of steatotic liver disease

	Men (*n* = 11 551)				Women (*n* = 6603)			
	Neither SLD (*n* = 7224)	NAFLD only (*n* = 60)	MASLD only (*n* = 1154)	Both SLDs (*n* = 3113)	Neither SLD (*n* = 5103)	NAFLD only (*n* = 36)	MASLD only (*n* = 74)	Both SLDs (*n* = 1390)
Prevalence (*N*, %)	191 (2.6%)	3 (5.0%)	60 (5.2%)	186 (6.0%)	47 (0.9%)	0 (0.0%)	4 (5.4%)	53 (3.8%)
Unadjusted OR	1 (reference)	1.94 (0.60–6.24)*	2.02 (1.50–2.72)	2.34 (1.90–2.88)	1 (reference)	0.00 (0.00)	6.15 (2.16–17.53)	4.26 (2.87–6.35)
Model 1	1 (reference)	2.65 (0.82–8.62)*	2.47 (1.82–3.34)	2.38 (1.93–2.94)	1 (reference)	0.00 (0.00)	5.08 (1.70–15.20)	3.01 (2.01–4.52)
Model 2	1 (reference)	2.60 (0.80–8.47)*	2.45 (1.81–3.32)	2.36 (1.91–2.90)	1 (reference)	0.00 (0.00)	4.97 (1.64–15.02)	2.97 (1.99–4.48)
Model 3	1 (reference)	2.67 (0.82–8.71)*	2.43 (1.79–3.30)	2.41 (1.95–2.98)	1 (reference)	0.00 (0.00)	4.91 (1.63–14.82)	3.02 (2.01–4.55)
Model 4	1 (reference)	2.66 (0.82–8.69)*	2.38 (1.74–3.25)	2.37 (1.91–2.95)	1 (reference)	0.00 (0.00)	4.72 (1.55–14.36)	2.88 (1.85–4.49)

*Note*: Data are presented as ORs (95% confidence intervals) unless otherwise indicated. Model 1 was adjusted for age. Model 2 was adjusted for age, smoking and exercise. Model 3 was adjusted for age, smoking, exercise, estimated glomerular filtration rate (eGFR) and high‐sensitivity C‐reactive protein (hsCRP). Model 4 was adjusted for age, smoking, exercise, eGFR, hsCRP and visceral fat area/subcutaneous fat area. Abbreviations: MASLD, metabolic dysfunction‐associated steatotic liver disease; NAFLD, non‐alcoholic fatty liver disease; OR, odds ratio; SLD, steatotic liver disease.

* *P* > 0.05; otherwise, all *P* < 0.05.

In those with NAFLD only, the ORs for sarcopenia were not significantly increased, with multivariable‐adjusted ORs (95% CI) of 2.66 (0.82–8.69) in men and 0.00 (0.00) in women (*Table* *2*, Model 4).

### Risk of myosteatosis according to each steatotic liver disease group

We also examined the prevalence and adjusted ORs for having myosteatosis across the SLD groups (*Table* *3*). Subjects with MASLD only and both SLDs demonstrated a significantly higher prevalence of myosteatosis (29.6% and 28.3% in men and 59.5% and 61.3% in women, respectively) than those with NAFLD only (11.7% in men and 19.4% in women). Those with MASLD also showed a higher association with myosteatosis, with multivariable‐adjusted ORs (95% CIs) of 1.69 (1.46–1.96) in those with MASLD only and 1.48 (1.33–1.64) in those with both SLDs among men and 1.83 (1.09–3.07) in those with MASLD only and 1.58 (1.37–1.83) in those with both SLDs among women (*Table* *3*, Model 4).

**Table 3:** Prevalence and odds ratios for having myosteatosis according to the presence of steatotic liver disease

	Men (*n* = 11 551)				Women (*n* = 6603)			
	Neither SLD (*n* = 7224)	NAFLD only (*n* = 60)	MASLD only (*n* = 1154)	Both SLDs (*n* = 3113)	Neither SLD (*n* = 5103)	NAFLD only (*n* = 36)	MASLD only (*n* = 74)	Both SLDs (*n* = 1390)
Prevalence (*N*, %)	1452 (20.1%)	7 (11.7%)	342 (29.6%)	881 (28.3%)	1858 (36.4%)	7 (19.4%)	44 (59.5%)	852 (61.3%)
Unadjusted OR	1 (reference)	0.53 (0.24–1.16)*	1.67 (1.46–1.92)	1.57 (1.42–1.73)	1 (reference)	0.42 (0.18–0.96)	2.56 (1.61–4.10)	2.78 (2.45–3.13)
Model 1	1 (reference)	0.65 (0.29–1.46)*	1.99 (1.72–2.30)	1.61 (1.45–1.78)	1 (reference)	0.45 (0.19–1.07)*	2.40 (1.45–3.98)	2.10 (1.84–2.39)
Model 2	1 (reference)	0.68 (0.30–1.49)*	1.93 (1.67–2.23)	1.59 (1.44–1.76)	1 (reference)	0.44 (0.19–1.04)*	2.37 (1.43–3.92)	2.08 (1.83–2.37)
Model 3	1 (reference)	0.67 (0.30–1.50)*	1.92 (1.66–2.22)	1.63 (1.47–1.80)	1 (reference)	0.41 (0.17–0.98)	2.32 (1.39–3.88)	2.02 (1.77–2.30)
Model 4	1 (reference)	0.66 (0.29–1.49)*	1.69 (1.46–1.96)	1.48 (1.33–1.64)	1 (reference)	0.38 (0.16–0.91)	1.83 (1.09–3.07)	1.58 (1.37–1.83)

*Note*: Data are presented as ORs (95% confidence intervals) unless otherwise indicated. Model 1 was adjusted for age. Model 2 was adjusted for age, smoking and exercise. Model 3 was adjusted for age, smoking, exercise, estimated glomerular filtration rate (eGFR) and high‐sensitivity C‐reactive protein (hsCRP). Model 4 was adjusted for age, smoking, exercise, eGFR, hsCRP and visceral fat area/subcutaneous fat area. Abbreviations: MASLD, metabolic dysfunction‐associated steatotic liver disease; NAFLD, non‐alcoholic fatty liver disease; OR, odds ratio; SLD, steatotic liver disease.

* *P* > 0.05; otherwise, all *P* < 0.05.

In subjects with NAFLD only, the multivariable‐adjusted ORs (95% CI) were 0.67 (0.30–1.50) in men and 0.41 (0.17–0.98) in women (*Table* *3*, Model 3). When the values were further adjusted with the visceral fat to subcutaneous fat ratio, the ORs (95% CI) were 0.66 (0.29–1.49) in men and 0.38 (0.16–0.91) in women (*Table* *3*, Model 4).

**Figure 3:**
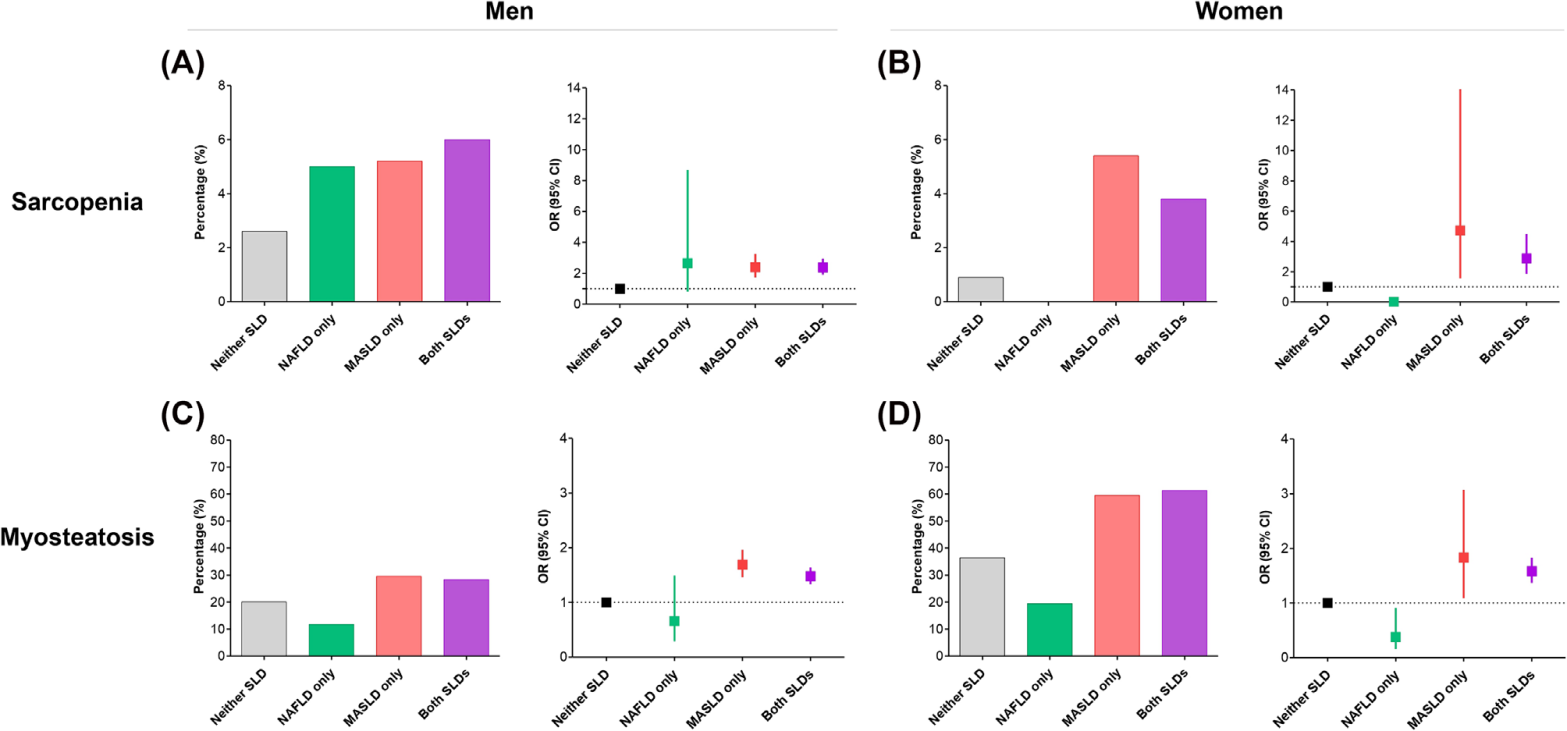
Summarized figure for the association between steatotic liver disease (SLD) and muscle mass and quality. (A) Prevalence and (B) odds ratios (ORs) for having sarcopenia according to the presence of SLD. (C) Prevalence and (D) ORs for having myosteatosis according to the presence of SLD. The ORs were adjusted for age, smoking, exercise, estimated glomerular filtration rate, high‐sensitivity C‐reactive protein and visceral fat area/subcutaneous fat area. CI, confidence interval; MASLD, metabolic dysfunction‐associated steatotic liver disease; NAFLD, non‐alcoholic fatty liver disease.

A summarized figure illustrating the association between SLD subtypes and muscle mass and quality for the entire cohort is presented in *Figure* *3*.

### Subgroup analysis

Among the participants with MASLD (*n* = 5731), 4979 were overweight or obese, 3616 had abdominal obesity and 1128 had diabetes mellitus (*Table* *4*). In subjects with MASLD, the presence of general overweight or obesity had a significant effect on the ORs for subjects with sarcopenia and myosteatosis. Subjects with MASLD who were under BMI 23 kg/m^2^ displayed insignificant associations with sarcopenia and myosteatosis (adjusted ORs [95% CIs] of 0.54 [0.28–1.02] for sarcopenia and 0.85 [0.71–1.01] for myosteatosis).

**Table 4:** Subgroup analysis according to the presence of overweight/obesity, abdominal obesity and diabetes mellitus

	Neither SLD	MASLD (*n* = 5731)					
		Overweight or obesity		Abdominal obesity		Diabetes mellitus	
		No	Yes	No	Yes	No	Yes
Total *N*	12 327	752	4979	2115	3616	4603	1128
Sarcopenia							
Prevalence	238 (1.9%)	10 (1.3%)	293 (5.9%)	83 (3.9%)	220 (6.1%)	210 (4.6%)	93 (8.2%)
Crude ORs	1 (reference)	0.69 (0.36–1.29)*	3.18 (2.67–3.78)	2.08 (1.61–2.68)	3.29 (2.73–3.97)	2.43 (2.01–2.93)	4.56 (3.56–5.85)
Adjusted ORs^a^	1 (reference)	0.54 (0.28–1.02)*	2.73 (2.26–3.28)	1.84 (1.41–2.40)	2.66 (2.19–3.25)	2.20 (1.81–2.69)	3.00 (2.30–3.90)
Myosteatosis							
Prevalence	3310 (26.9%)	216 (28.7%)	1903 (38.2%)	386 (18.3%)	1733 (47.9%)	1634 (35.5%)	485 (43.0%)
Crude ORs	1 (reference)	1.10 (0.93–1.29)*	1.69 (1.57–1.81)	0.61 (0.54–0.68)	2.51 (2.32–2.71)	1.50 (1.40–1.61)	2.06 (1.81–2.33)
Adjusted ORs^a^	1 (reference)	0.85 (0.71–1.01)*	1.92 (1.77–2.08)	0.65 (0.57–0.74)	2.65 (2.43–2.90)	1.69 (1.56–1.84)	1.85 (1.61–2.12)

*Note*: Data are presented as ORs (95% confidence intervals) unless otherwise indicated. Abbreviations: MASLD, metabolic dysfunction‐associated steatotic liver disease; ORs, odds ratios; SLD, steatotic liver disease.

^a^ ORs were adjusted for age, smoking, exercise, estimated glomerular filtration rate, high‐sensitivity C‐reactive protein and visceral fat area/subcutaneous fat area.

* *P* > 0.05; otherwise, all *P* < 0.05.

On the contrary, the presence of abdominal obesity had a significant impact on the association with myosteatosis but not with sarcopenia (*Table* *4*).

## Discussion

In this large cross‐sectional study, we analysed the association between the newly proposed concept, MASLD, and muscle characteristics, including muscle mass and quality. The results revealed a higher association between both sarcopenia and myosteatosis and the existence of MASLD, independent of the presence of NAFLD. The results were still significant after adjusting for various metabolic risk factors. Adopting the MASLD concept proved to be more effective in identifying individuals with reduced muscle mass and compromised muscle quality than relying solely on the NAFLD classification.

Although most prior observational studies have reported a positive correlation between sarcopenia and NAFLD,^20^, ^21^ there have been reports of a relatively low prevalence of sarcopenia in individuals with NAFLD.^22^ One study even failed to demonstrate a significant correlation between the amount of hepatic fat and the presence of myosteatosis or sarcopenia in individuals with NAFLD.^23^ While no studies have explored the association with sarcopenia following the introduction of the MASLD term, several studies have demonstrated a strong correlation between conditions previously termed MAFLD (metabolic dysfunction‐associated fatty liver disease) and decreased muscle mass.^24^, ^25^, ^26^ Research on the prevalence of myosteatosis in MASLD is rare, despite its more robust association with fibrosis progression than sarcopenia.^27^ To the best of our knowledge, our study is the first to investigate the association between MASLD and myosteatosis and to simultaneously evaluate the quantity and quality of muscle.

According to the traditional NAFLD definition, nearly 40% of people with NAFLD are not obese.^28^ Previous research has consistently shown a positive association between sarcopenia and these non‐obese subjects with NAFLD,^20^, ^21^, ^29^ irrespective of other metabolic syndromes. The pathogenesis of non‐obese NAFLD has been largely explained by central obesity, as indicated by a more visceral fat area, along with other factors such as type 2 diabetes mellitus, chronic inflammation and/or a high‐caloric diet.^30^ Most of these factors are now incorporated into the new diagnostic criteria for MASLD.^1^ Consequently, the NAFLD‐only group in our study represents a more specific population than previously categorized as non‐obese NAFLD. Indeed, our NAFLD‐only cohort exhibited a lower visceral fat area compared with that of the MASLD group (*Table* *1*), and the severity of SLD was relatively mild (*Table* *S2*), potentially explaining the lack of impact on skeletal muscle attributes (*Tables* *2* and *3*). The limited number of subjects in the NAFLD‐only group in this study (0.5%, 96 out of 18 154) also made it difficult to draw a conclusion on this issue.

MASLD also involves heterogeneous populations, depending on the accompanying metabolic dysfunctions.^31^ In our subgroup analysis, we found that non‐obese individuals with MASLD showed comparable risks of sarcopenia and myosteatosis to those without SLD. On the contrary, while the presence of abdominal obesity seems to have a notable impact on the association of MASLD with myosteatosis, it does not appear to influence the occurrence of sarcopenia. The pathogenesis of MASLD in non‐obese individuals remains enigmatic, raising questions about why certain non‐obese individuals develop SLD. Genetic polymorphisms, particularly those involving the patatin‐like phospholipase domain‐containing 3 (PNPLA3) gene, have been suggested as potential mechanisms.^32^ Genetic variations in PNPLA3 are known to increase hepatic TG accumulation and susceptibility to steatosis^33^ and influence the development of SLD in non‐obese individuals without metabolic syndrome^34^, ^35^; however, further investigations are needed to explore the impact of these genetic factors on skeletal muscle and the long‐term metabolic consequences specific to this population.

According to the revised nomenclature and diagnostic criteria for fatty liver disease proposed by the American Association for the Study of Liver Diseases (AASLD) and the European Association for the Study of the Liver (EASL),^1^ the acronym MetALD was introduced to define a distinct patient subgroup with higher alcohol intake among patients with MASLD who consume 140–350 g/week (20–50 g/day) for females and 210–420 g/week (30–60 g/day) for males. We conducted an additional analysis by designating a strict MASLD group, which did not include patients with MetALD (*Tables* *S3* and *S4* and *Figure* *S2*). Upon excluding patients meeting the MetALD criteria, the results were not statistically significant due to the limited number of patients in each subgroup.

Bioelectrical impedance analysis is a guideline‐accepted method for detecting sarcopenia in the Asian population.^36^ Although there is ongoing research on the use of CT to diagnose sarcopenia, standardized CT thresholds for diagnosing sarcopenia remain unclear.^37^ Among the various CT‐measured skeletal muscle indices, skeletal muscle area (SMA) adjusted with BMI (i.e., SMA/BMI) has been proposed to be an ideal index for diagnosing sarcopenia due to its high diagnostic accuracy and strong correlation with age‐related muscle loss patterns.^38^ When the association between SLD and sarcopenia was additionally analysed using SMA/BMI, the results were generally consistent with those obtained using the bioelectrical impedance analysis‐based diagnosis (*Table* *S5*).

In subjects with NAFLD, it has been observed that hepatic steatosis is more closely linked to skeletal muscle‐induced insulin resistance than hepatic insulin resistance, supporting the critical role of skeletal muscle in the development of SLD.^39^ Given the shared pathophysiology between SLD and skeletal muscle‐induced insulin resistance, it is difficult to evaluate whether sarcopenia and myosteatosis are fundamental causes of MASLD development or consequences thereof. The deterioration of muscle energy metabolism influences SLD through mechanisms such as insulin resistance, chronic inflammation, increased hepatic oxidative stress and exacerbated fibrosis.^40^ When fat infiltrates skeletal muscle, it induces mitochondrial dysfunction and impaired insulin signalling, while insulin resistance exacerbates proteolysis of muscle, further contributing to muscle loss.^20^ These alterations affect the secretion of myokines, which result in disruption of hepatic glucose homeostasis, increased hepatic free fatty acid uptake, reduced fatty acid oxidation and ultimately aggravated hepatic steatosis and fibrosis.^20^, ^40^

There are several limitations to our study. First, we could not prove a causal relationship between SLD and muscle outcomes because of the cross‐sectional design of the study. Second, the fibrotic stage of SLD within the study cohort could not be determined. Third, our assessment of sarcopenia focused on muscle mass and did not consider muscle strength. Fourth, the dataset was collected a decade ago and may not reflect changes in the prevalence of metabolic risk factors over the period; hence, future research using more recent data is warranted. Fifth, in our study, the diagnosis of SLD relied on abdominal ultrasound without pathological confirmation through liver biopsy. The specificity of this diagnostic technique is relatively low, as it can detect hepatic steatosis only when it exceeds 25–30%,^41^ and the sensitivity varies depending on SLD severity.^42^ However, when the patients were stratified by steatosis severity, similar findings were observed in the mild SLD group as in the moderate to severe SLD group (data now shown). Sixth, we were unable to establish the treatment effect of MASLD on muscle characteristics. Given the increasing prevalence of MASLD as a cause of liver cirrhosis and hepatocellular carcinoma (HCC) and considering that tyrosine kinase inhibitors used in HCC treatment can exacerbate muscle loss,^43^ exploring treatment strategies in MASLD patients warrants clinical attention and further investigation in future studies. Finally, our study sample consisted of Korean individuals who participated in routine health checkups, limiting the generalizability of our results to other populations or ethnicities.

In conclusion, our study highlights the increased association of sarcopenia and myosteatosis in participants with MASLD. Our study findings support the implementation of the term MASLD in place of NAFLD, as it demonstrated a stronger predictive value for adverse muscle characteristics than NAFLD. Because MASLD places more emphasis on the metabolic aspect, it may enhance multidisciplinary efforts to manage this complex condition. Future studies on the effect of therapeutic interventions to improve hepatic steatosis in those with MASLD are warranted.

## Conflict of interest statement

The authors declare that they have no competing interests.

## Funding information

The author(s) received no financial support for the research, authorship and/or publication of this article.

## Supporting information


**Table S1.** Baseline characteristics and CT measurements of the participants by the presence of steatotic liver disease (SLD).
**Table S2.** SLD severity of the participants according to the SLD groups.
**Table S3.** Baseline characteristics and CT measurements of the participants by the presence of SLD (excluding those meeting MetALD criteria).
**Table S4.** Prevalence and ORs for having sarcopenia and myosteatosis according to the presence of SLD (excluding those meeting MetALD criteria).
**Table S5.** Prevalence and ORs for having sarcopenia, defined by CT‐derived definition.
**Figure S1.** Flow diagram of study participants
**Figure S2.** Grouping of study participants according to the presence of SLD (excluding those meeting MetALD criteria) (*N* = 17,108).
