# Supplementary material for: Association between metabolic dysfunction‐associated steatotic liver disease and myosteatosis measured by computed tomography
Source: J Cachexia Sarcopenia Muscle. 2024 Jul 16;15(5):1942–52. doi: 10.1002/jcsm.13543 (PMC11446687; doi:10.1002/jcsm.13543)
Supplement: Supplementary file 1 — Table S1. Baseline characteristics and CT measurements of the participants by the presence of steatotic liver disease (SLD). Table S2. SLD severity of the participants according to the SLD groups. Table S3. Baseline characteristics and CT measurements of the participants by the presence of SLD (excluding those meeting MetALD criteria). Table S4. Prevalence and ORs for having sarcopenia and myosteatosis according to the presence of SLD (excluding those meeting MetALD criteria). Table S5. Prevalence and ORs for having sarcopenia, defined by CT‐derived definition. Figure S1. Flow diagram of study participants Figure S2. Grouping of study participants according to the presence of SLD (excluding those meeting MetALD criteria) (N = 17,108). [file JCSM-15-1942-s001.docx]

**SUPPLEMENTARY MATERIALS**

**Contents**

**Supplementary Methods**

**1. Assessments on laboratory and anthropometric data**

**2. Acquisition of computed tomography (CT) imaging**

**Table S1. Baseline characteristics and CT measurements of the participants by the presence of steatotic liver disease (SLD).**

**Table S2. SLD severity of the participants according to the SLD groups.**

**Table S3. Baseline characteristics and CT measurements of the participants by the presence of SLD (excluding those meeting MetALD criteria).**

**Table S4. Prevalence and ORs for having sarcopenia and myosteatosis according to the presence of SLD (excluding those meeting MetALD criteria).**

**Table S5. Prevalence and ORs for having sarcopenia, defined by CT-derived definition.**

**Figure S1. Flow diagram of study participants**

**Figure S2. Grouping of study participants according to the presence of SLD (excluding those meeting MetALD criteria) (N = 17,108).**

**Supplementary Methods**

**1. Assessments on laboratory and anthropometric data**

The waist circumference, height, and weight were measured by standardized procedures. Body mass index (BMI) was derived by dividing weight in kilograms by height in meters squared. Blood pressure (BP) was checked with an automatic manometer on the right arm after taking at least five minutes of rest. Hypertension was defined as a systolic/diastolic BP of 140/90 mmHg or continued antihypertensive medication. Body composition was measured with direct segmental multi-frequency bioelectrical impedance analysis using InBody 720 (InBody CO., Ltd, Seoul, Korea). The system separately measured the impedance of each participant’s right arm, left arm, trunk, right leg and left leg at six different frequencies (1, 5, 50, 250, 500 and 1000 kHz).

The blood sample was collected the morning following overnight fasting. Fasting total cholesterol, high-density lipoprotein (HDL) cholesterol, low-density lipoprotein cholesterol (LDL-C), triglycerides (TG), uric acid, aspartate aminotransferase (AST), and alanine aminotransferase (ALT) levels were measured with enzymatic colorimetric methods using a Toshiba 200FR Neo analyzer (Toshiba Medical System Co., Ltd., Tokyo, Japan). Gamma-glutamyl transferase (GGT) levels were measured using the L-γ-glutamyl-p-nitroanilide method (Toshiba). Creatinine levels were measured using the Jaffe method. Fasting plasma glucose (FPG) was measured with the hexokinase method, and high-sensitivity C-reactive protein (hsCRP) was measured with the immunoturbidimetric method using a Toshiba 200FR Neo analyzer (Toshiba Medical System Co., Ltd., Tokyo, Japan). Ion-exchange high-performance liquid chromatography (Bio-Rad Laboratories, Inc, Hercules, CA) was used to measure the glycated hemoglobin (HbA1c) levels. All enzymatic activities were analyzed at 37°C.

**2. Acquisition of computed tomography (CT) imaging**

Somatom Definition (Siemens Healthineers, Erlangen, Germany), Discovery CT750 HD (GE Healthcare, Milwaukee, WI, USA), and LightSpeed VCT scanners (GE Healthcare) were used for the abdomen and pelvic CT scans. The following parameters were applied in all CT examinations: 120 kVp; automatic dose modulation (CareDose 4D, Siemens Healthineers; automA and smartmA, GE Healthcare); matrix 512 × 512; collimation of 0.625 mm. Image reconstruction was performed with a 5 mm slice thickness via the filtered back-projection approach and a soft tissue reconstruction algorithm (B30f kernel; Siemens Healthineers; Standard kernel, GE Healthcare). For contrast-enhanced scans, 100–150 mL of iopromide (Ultravist 370 or Ultravist 300; Bayer Schering Pharma, Berlin, Germany) was injected intravenously at a speed of 2.5–3 mL/s using an automated power injector. Contrast administration was followed by a fixed scan delay of 70 seconds.

**Table S1. Baseline characteristics and CT measurements of the participants by the presence of steatotic liver disease (SLD).**

|  | **NAFLD** | | **P** | **MASLD** | | **P** |
| --- | --- | --- | --- | --- | --- | --- |
|  | No (n=13,555) | Yes (n=4,599) |  | No (n=12,423) | Yes (n=5,731) |  |
| Age (years) | 52.6±8.8 | 54.2±8.8 | <0.001 | 52.7±8.9 | 53.7±8.6 | <0.001 |
| Body mass index (kg/m^2^) | 23.2±2.8 | 25.6±2.9 | <0.001 | 23.0±2.6 | 25.8±2.8 | <0.001 |
| Waist circumference (cm) | 82.4±8.6 | 89.4±7.7 | <0.001 | 81.5±8.2 | 90.0±7.6 | <0.001 |
| Systolic BP (mmHg) | 120.8±14.1 | 125.7±13.5 | <0.001 | 120.0±14.0 | 126.5±13.4 | <0.001 |
| Diastolic BP (mmHg) | 76.8±10.8 | 79.8±10.3 | <0.001 | 76.1±10.7 | 80.7±10.3 | <0.001 |
| Current smoker (%) | 22.5 | 24.4 | <0.001 | 21.1 | 26.9 | <0.001 |
| Excess drinking (%) | 28.7 | 0.0 | <0.001 | 22.9 | 18.3 | <0.001 |
| Physically active (%) | 45.1 | 40.6 | <0.001 | 45.9 | 39.9 | <0.001 |
| Obesity (BMI>25 kg/m^2^) (%) | 25.3 | 54.6 | <0.001 | 47.7 | 86.9 | <0.001 |
| Hypertension (%) | 37.6 | 53.3 | <0.001 | 64.7 | 56.5 | <0.001 |
| Dyslipidemia therapy (%) | 10.0 | 16.7 | <0.001 | 9.5 | 16.5 | <0.001 |
| Family history of diabetes (%) | 21.5 | 25.2 | <0.001 | 20.7 | 26.0 | <0.001 |
| FPG (mg/dL) | 98.3±15.9 | 105.6±22.4 | <0.001 | 97.4±14.8 | 106.5±22.5 | <0.001 |
| HbA1c (%) | 5.54±0.58 | 5.90±0.84 | <0.001 | 5.51±0.54 | 5.90±0.84 | <0.001 |
| Total cholesterol (mg/dL) | 194.0±33.6 | 198.4±36.8 | <0.001 | 193.6±33.2 | 198.4±36.9 | <0.001 |
| TG (mg/dL) | 93 (68-131) | 128 (95-175) | <0.001 | 90 (67-124) | 132 (98-181) | <0.001 |
| LDL-C (mg/dL) | 121.2±30.0 | 128.7±32.6 | <0.001 | 120.7±29.7 | 128.3±32.7 | <0.001 |
| HDL-C (mg/dL) | 57.6±14.7 | 48.6±11.5 | <0.001 | 58.4±14.7 | 48.7±11.6 | <0.001 |
| Uric acid (mg/dL) | 5.24±1.38 | 5.76±1.37 | <0.001 | 5.15±1.34 | 5.86±1.39 | <0.001 |
| AST (U/L) | 28.2±18.9 | 29.5±13.6 | <0.001 | 27.0±14.4 | 30.5±21.4 | <0.001 |
| ALT (U/L) | 27.6±18.0 | 32.8±21.9 | <0.001 | 22.6±17.4 | 33.7±25.3 | <0.001 |
| GGT (U/L) | 30.8±42.7 | 33.9±30.8 | <0.001 | 28.2±37.2 | 39.0±44.8 | <0.001 |
| hsCRP (mg/L) | 0.04 (0.02-0.09) | 0.07 (0.04-0.14) | <0.001 | 0.02 (0.04-0.08) | 0.07 (0.04-0.14) | <0.001 |
| HOMA-IR | 0.95 (0.56-1.51) | 1.67 (1.11-2.41) | <0.001 | 0.90 (0.54-1.41) | 1.70 (1.14-2.42) | <0.001 |
| Body fat mass, kg | 15.2±5.0 | 19.3±5.7 | <0.001 | 14.8±4.7 | 19.3±5.7 | <0.001 |
| Skeletal muscle mass, kg | 27.3±5.8 | 28.9±5.8 | <0.001 | 26.8±5.7 | 29.6±5.8 | <0.001 |
| Appendicular skeletal muscle, kg | 20.6±4.6 | 21.8±4.5 | <0.001 | 20.3±4.5 | 22.3±4.5 | <0.001 |
| Appendicular skeletal muscle /BMI, m^2^ | 0.89±0.17 | 0.76±0.10 | <0.001 | 0.88±0.17 | 0.87±0.16 | <0.001 |
| Visceral fat area/subcutaneous fat area | 0.83±0.50 | 1.10±0.52 | <0.001 | 0.78±0.47 | 1.15±0.52 | <0.001 |
| TAMA, cm^2^ | 145.5±33.2 | 156.8±32.9 | <0.001 | 142.5±32.3 | 161.1±32.3 | <0.001 |
| NAMA, cm^2^ | 111.7±30.0 | 116.8±31.3 | <0.001 | 109.6±29.7 | 120.3±30.6 | <0.001 |
| LAMA, cm^2^ | 28.6±10.7 | 33.5±11.6 | <0.001 | 27.8±10.2 | 34.3±11.8 | <0.001 |
| IMAT, cm^2^ | 5.24±4.03 | 6.46±5.10 | <0.001 | 5.12±3.96 | 6.47±5.02 | <0.001 |
| NAMA/TAMA index | 76.4±8.6 | 73.9±9.4 | <0.001 | 76.5±8.6 | 74.2±9.2 | <0.001 |

**Table S2.** **SLD severity of the participants according to the SLD groups.**

| **Fatty liver severity** | **Neither FLD**  (n=12,327) | **NAFLD only**  (n=96) | **MASLD only**  (n=1,228) | **Both FLDs**  (n=4,503) |
| --- | --- | --- | --- | --- |
| **No SLD** | 12,309 (99.9%) |  |  |  |
| **Mild** | 16 (0.1%) | 81 (84.4%) | 794 (64.7%) | 2,712 (60.2%) |
| **Moderate** | 2 (0.0%) | 15 (15.6%) | 358 (29.2%) | 1,450 (32.2%) |
| **Severe** | 0 | 0 (0.0%) | 75 (6.1%) | 341 (7.6%) |

**Table S3. Baseline characteristics and CT measurements of the participants by the presence of SLD (excluding those meeting MetALD criteria).**

|  | **Neither FLD** | **NAFLD only** | **MASLD only** | **Both FLDs** | **P** |
| --- | --- | --- | --- | --- | --- |
| N (%) | 12,397 (72.1%) | 96 (0.6%) | 182 (1.1%) | 4,503 (26.3%) |  |
| Age (years) | 52.7±8.9^a^ | 50.6±7.1^a^ | 52.8±8.4^ab^ | 54.2±8.8^b^ | <0.001 |
| Sex (Male, N [%]) | 7224 (58.6%) | 60 (62.5%) | 143 (78.6%) | 3,113 (69.1%) | <0.001 |
| Body mass index (kg/m^2^) | 23.0±2.6 | 21.6±1.0 | 25.8±2.8^a^ | 25.7±2.8^a^ | <0.001 |
| Waist circumference (cm) | 81.5±8.2^a^ | 79.5±5.1^a^ | 90.3±7.3^b^ | 89.6±7.6^b^ | <0.001 |
| Systolic BP (mmHg) | 120.0±14.0 | 114.6±8.1 | 127.2±13.8^a^ | 126.0±13.5^a^ | <0.001 |
| Diastolic BP (mmHg) | 76.1±10.7 | 72.6±6.9 | 80.8±10.2^a^ | 80.0±10.3^a^ | <0.001 |
| Current smoker (%) | 21.2 | 20.0 | 26.4 | 24.4 | <0.001 |
| Excess drinking (%) | 23.1 | 0.0 | 0.0 | 0.0 | <0.001 |
| Physically active (%) | 46.0 | 33.3 | 36.8 | 40.8 | <0.001 |
| Obesity (BMI>25 kg/m^2^) (%) | 21.4 | 0.0 | 61.5 | 55.7 | <0.001 |
| Hypertension (%) | 6.7 | 0.0 | 19.8 | 19.6 | <0.001 |
| Dyslipidemia therapy (%) | 9.6 | 0.0 | 9.9 | 17.1 | <0.001 |
| Family history of diabetes (%) | 20.7 | 20.8 | 25.3 | 25.2 | <0.001 |
| FPG (mg/dL) | 97.4±14.9 | 90.6±6.0 | 106.1±22.7^a^ | 99.8±17.8^a^ | <0.001 |
| HbA1c (%) | 5.51±0.54 | 5.32±0.21 | 5.85±0.85^a^ | 5.91±0.85^a^ | <0.001 |
| Total cholesterol (mg/dL) | 193.6±33.2^a^ | 198.4±28.6^ab^ | 193.2±34.6^ab^ | 198.3±37.0^ab^ | <0.001 |
| TG (mg/dL) | 90 (67–124) | 91 (76–113) | 122 (88–152) | 130 (96–179) | <0.001 |
| LDL-C (mg/dL) | 120.6±29.7 | 129.5±24.2^a^ | 127.2±30.8^a^ | 128.7±32.7^a^ | <0.001 |
| HDL-C (mg/dL) | 58.4±14.8^a^ | 55.2±9.3^a^ | 47.0±10.7^b^ | 48.4±11.5^b^ | <0.001 |
| Uric acid (mg/dL) | 5.15±1.34^a^ | 5.39±1.17^ab^ | 5.83±1.28^b^ | 5.77±1.38^b^ | <0.001 |
| AST (U/L) | 27.0±14.5^a^ | 25.9±7.2^a^ | 36.6±29.9^b^ | 29.6±13.7^b^ | <0.001 |
| ALT (U/L) | 22.6±17.4^a^ | 25.9±13.6^a^ | 39.8±71.5 | 33.0±22.1 | <0.001 |
| GGT (U/L) | 18 (13–30) | 19 (13–29) | 25 (18–41) | 26 (18–39) | <0.001 |
| hsCRP (mg/L) | 0.04 (0.02–0.08) | 0.04 (0.03–0.10) | 0.06 (0.03–0.14) | 0.07 (0.04–0.14) | <0.001 |
| HOMA-IR | 0.90 (0.54–1.41) | 0.87 (0.58–1.43) | 1.87 (1.42–2.53) | 1.69 (1.13–2.42) | <0.001 |
| Body fat mass, kg | 14.8±4.7^a^ | 13.7±2.5^a^ | 19.5±5.9^b^ | 19.4±5.7^b^ | <0.001 |
| Skeletal muscle mass, kg | 26.9±5.7^a^ | 25.7±4.8^a^ | 30.0±5.7^b^ | 29.0±5.9^b^ | <0.001 |
| Appendicular skeletal muscle, kg | 20.3±4.5 ^a^ | 19.6±4.1^a^ | 22.7±4.4^ab^ | 21.8±4.6^b^ | <0.001 |
| Appendicular skeletal muscle /BMI, m^2^ | 0.88±0.17^a^ | 0.91±0.18^a^ | 0.88±0.17 | 0.85±0.16 | <0.001 |
| Visceral fat area/subcutaneous fat area | 0.78±0.47^a^ | 0.83±0.39^a^ | 1.10±0.48^b^ | 1.11±0.52^b^ | <0.001 |
| TAMA, cm^2^ | 142.6±32.4^a^ | 134.9±27.0^a^ | 162.8±29.4^b^ | 157.2±32.9^b^ | <0.001 |
| NAMA, cm^2^ | 109.6±29.7^a^ | 106.2±25.4^a^ | 124.0±29.8 | 117.0±31.3 | <0.001 |
| LAMA, cm^2^ | 27.8±10.2 | 24.4±7.6 | 37.2±11.0^a^ | 33.7±11.6^a^ | <0.001 |
| IMAT, cm^2^ | 5.12±3.96^a^ | 4.28±3.20^a^ | 6.07±4.20^b^ | 6.49±5.14^b^ | <0.001 |
| NAMA/TAMA index | 76.5±8.6^a^ | 78.3±7.3^a^ | 75.6±9.3^ab^ | 73.8±9.4^b^ | <0.001 |

^a^The same superscripts imply a statistically insignificant difference between those values in post hoc analysis. Otherwise, post hoc analysis revealed significant differences between each group.

BP, blood pressures; BMI, body mass index; FPG, fasting plasma glucose; HbA1c, hemoglobin A1c; LDL-C, low-density lipoprotein cholesterol; HDL-C, high-density lipoprotein cholesterol; AST, aspartate aminotransferase; ALT, alanine aminotransferase; GGT, gamma-glutamyltransferase; hsCRP, high-sensitivity C-reactive protein; HOMA-IR, Homeostatic Model Assessment for Insulin Resistance; TAMA, total abdominal muscle area; NAMA, normal-attenuation muscle area; LAMA, low-attenuation muscle area; IMAT, intermuscular adipose tissue; NAFLD, non-alcoholic fatty liver disease; MAFLD, metabolic dysfunction-associated fatty liver disease; FLD, fatty liver disease.

**Table S4. Prevalence and ORs for having sarcopenia and myosteatosis according to the presence of SLD (****excluding those meeting MetALD criteria).**

|  | Neither FLD | NAFLD only | MASLD only | Both FLDs |
| --- | --- | --- | --- | --- |
|  | (n=12,397) | (n=96) | (n=182) | (n=4,503) |
| Sarcopenia |  |  |  |  |
| Prevalence (N, %) | 238 (1.9%) | 3 (3.1%) | 12 (5.5%) | 239 (5.3%) |
| Unadjusted | 1 (reference) | 1.64 (0.52–5.21) | 2.95 (1.54–5.66) | 2.85 (2.37–3.42) |
| Model 1 | 1 (reference) | 2.25 (0.70–7.20) | 3.13 (1.62–6.07) | 2.62 (2.17–3.15) |
| Model 2 | 1 (reference) | 2.24 (0.70–7.19) | 2.98 (1.54–5.79) | 2.55 (2.12–3.07) |
| Model 3 | 1 (reference) | 2.22 (0.69–7.11) | 2.98 (1.54–5.79) | 2.57 (2.13–3.09) |
| Model 4 | 1 (reference) | 2.17 (0.67–6.99) | 2.86 (1.56–5.26) | 2.30 (1.90–2.79) |
| Myostetosis |  |  |  |  |
| Prevalence (N, %) | 3,310 (26.9%) | 14 (14.6%) | 75 (34.6%) | 1,733 (38.5%) |
| Unadjusted | 1 (reference) | 0.47 (0.26–0.82) | 1.12 (0.81–1.55) | 1.70 (1.59–1.83) |
| Model 1 | 1 (reference) | 0.55 (0.31–0.99) | 1.14 (0.81–1.59) | 1.59 (1.48–1.72) |
| Model 2 | 1 (reference) | 0.55 (0.30–0.98) | 1.19 (1.85–1.68) | 1.66 (1.54–1.80) |
| Model 3 | 1 (reference) | 0.52 (0.29–0.95) | 1.21 (0.85–1.71) | 1.69 (1.57–1.83) |
| Model 4 | 1 (reference) | 0.52 (0.29–0.95) | 1.21 (0.86–1.72) | 1.71 (1.57–1.85) |

Data are presented as ORs (95% CIs) unless otherwise indicated.

Model 1 was adjusted for age.

Model 2 was adjusted for age, smoking, and exercise.

Model 3 was adjusted for age, smoking, exercise, eGFR, and hsCRP.

Model 4 was adjusted for age, smoking, exercise, eGFR, hsCRP, and visceral fat area/subcutaneous fat area.

NAFLD, non-alcoholic fatty liver disease; MASLD, metabolic dysfunction-associated steatotic liver disease; SLD, steatotic liver disease.

**Table S5. Prevalence and ORs for having sarcopenia, defined by CT-derived definition.**

|  | Neither SLD | NAFLD only | MASLD only | | Both SLDs |
| --- | --- | --- | --- | --- | --- |
|  | (n=12,327) | (n=96) | (n=1,228) | | (n=4,503) |
| CT-based sarcopenia (adopting SMA/BMI) | | | |  | |
| Prevalence (N, %) | 66 (0.5%) | 0 (0.0%) | 12 (1.0%) | | 66 (1.5%) |
| Unadjusted OR | 1 (reference) | 0.0 (0.0) | 1.83 (0.99–3.40) | | 2.76 (1.96–3.89) |
| Adjusted OR* | 1 (reference) | 0.0 (0.0) | 2.74 (1.42–5.27) | | 2.57 (1.78–3.71) |

*Adjustment variables included age, sex, smoking, exercise, eGFR, hsCRP, and visceral fat area/subcutaneous fat area

**Figure S1. An example of body composition analysis from an axial CT slice and a schematic diagram of segmentation.**


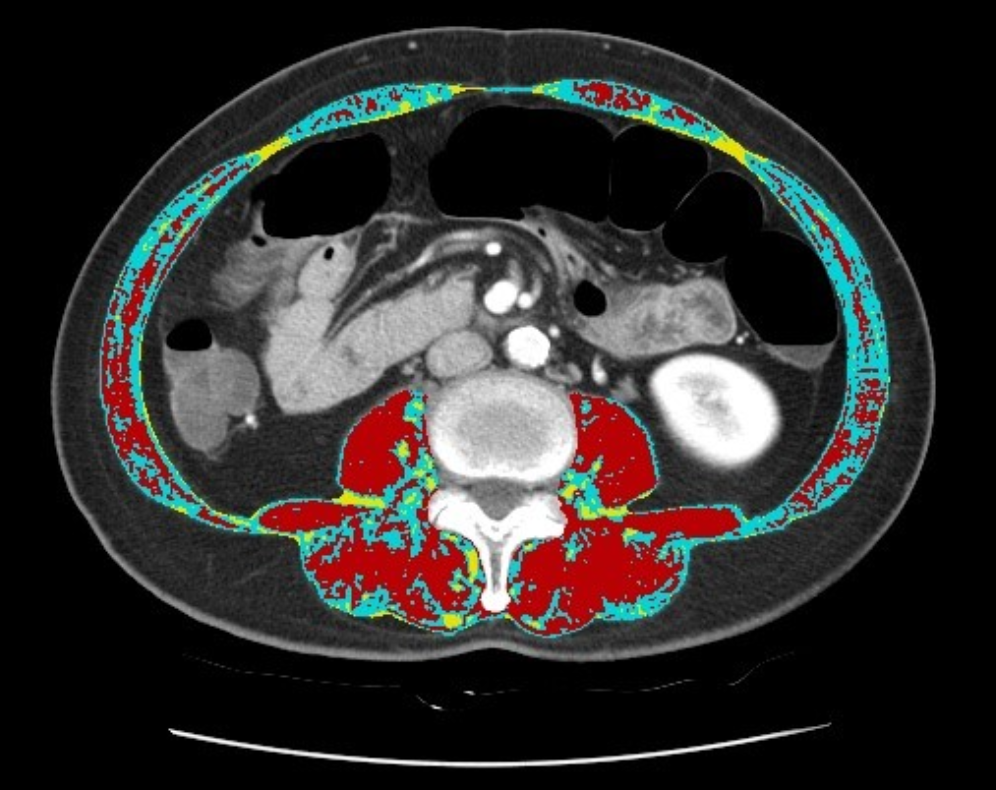

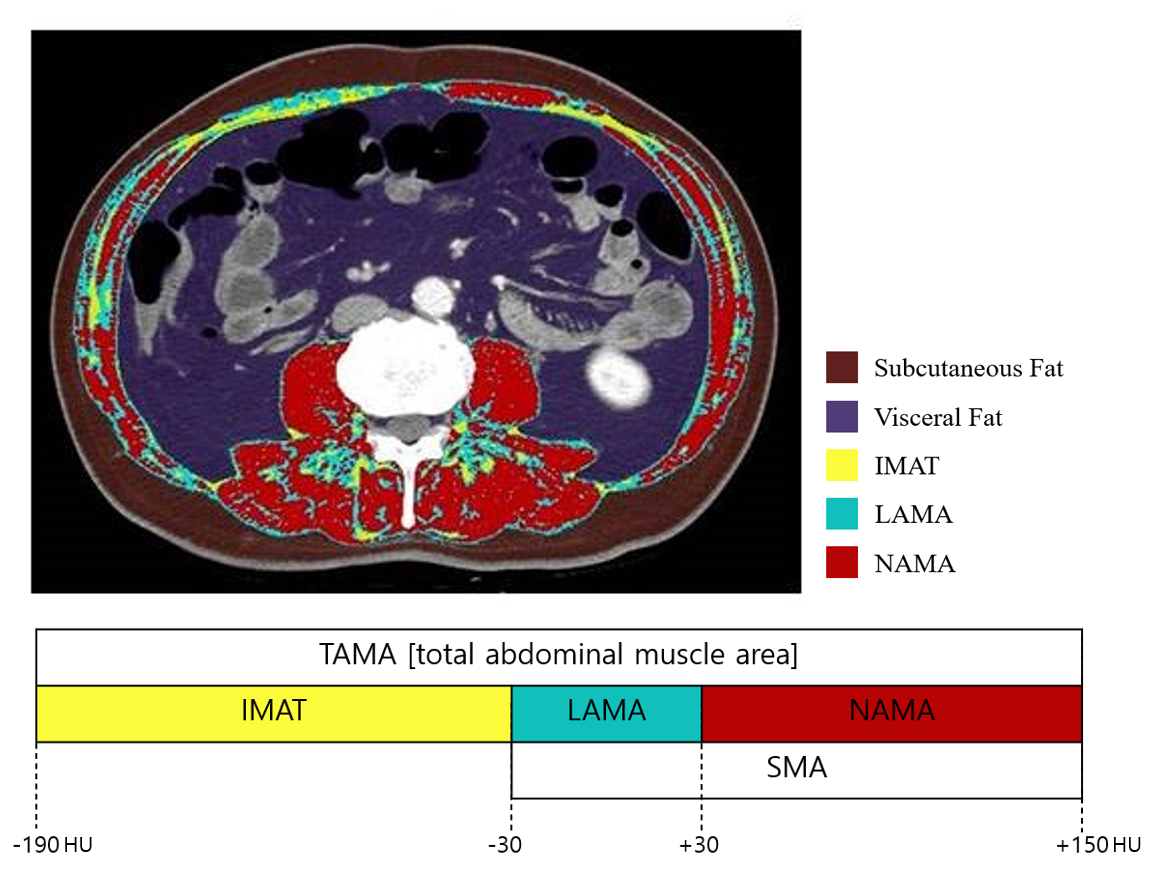

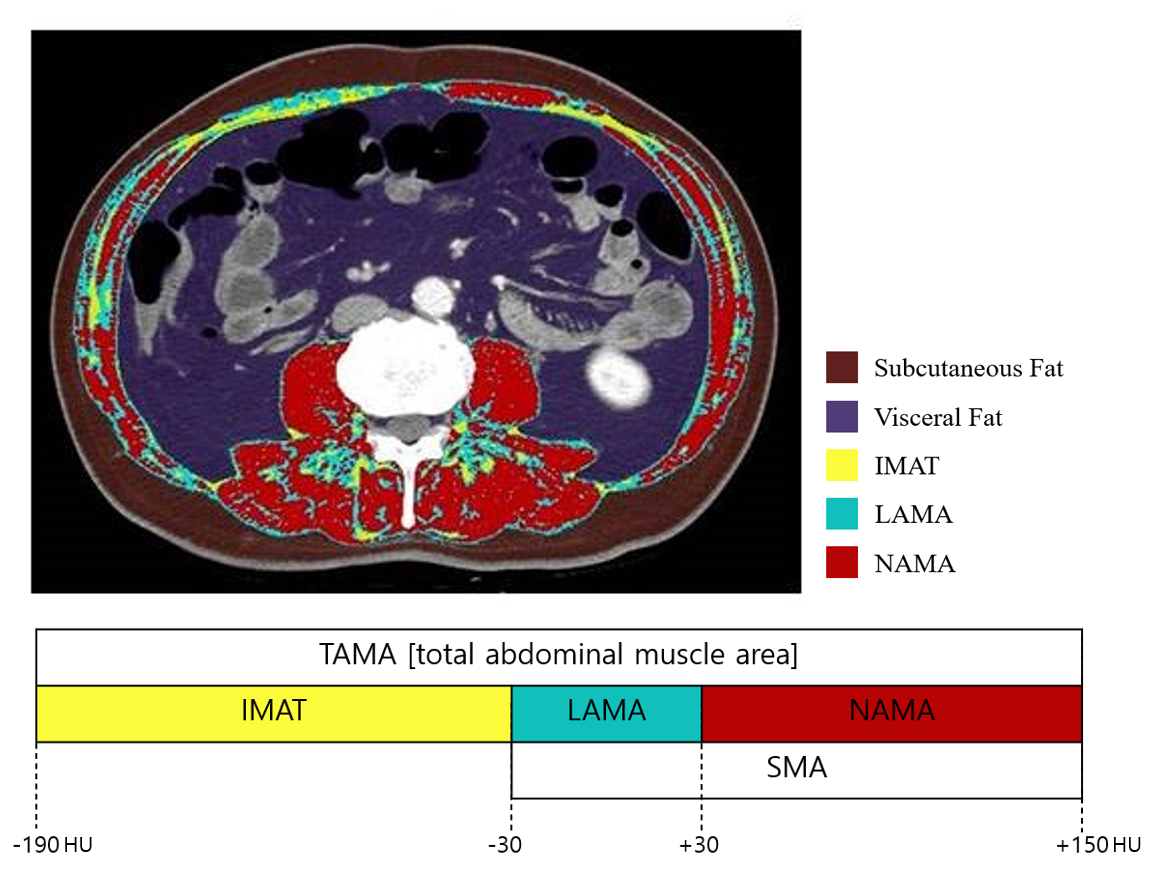


IMAT, intermuscular adipose tissue; LAMA, low attenuation muscle area; NAMA, normal attenuation muscle area; SMA, skeletal muscle area; HU, Hounsfield unit.

**Figure S2. Grouping of study participants according to the presence of SLD (excluding those meeting MetALD criteria) (N = 17,108).**

**
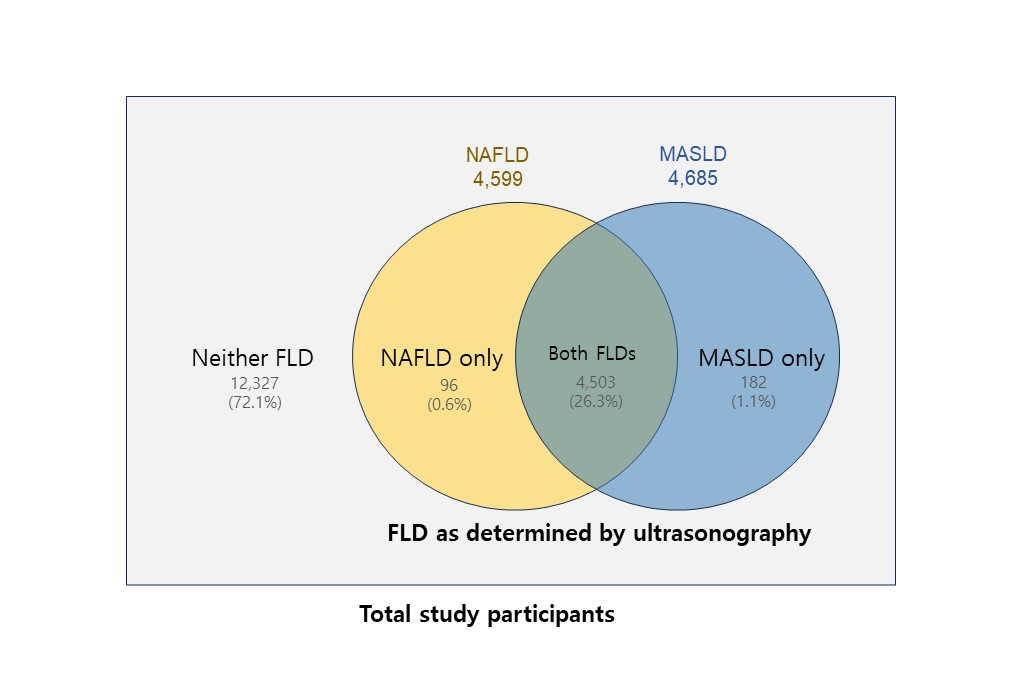
**
